# The first complete mitochondrial genome of *Dacus longicornis* (Diptera: Tephritidae) using next-generation sequencing and mitochondrial genome phylogeny of Dacini tribe

**DOI:** 10.1038/srep36426

**Published:** 2016-11-04

**Authors:** Fan Jiang, Xubin Pan, Xuankun Li, Yanxue Yu, Junhua Zhang, Hongshan Jiang, Liduo Dou, Shuifang Zhu

**Affiliations:** 1 Institute of Plant Quarantine, Chinese Academy of Inspection and Quarantine, Beijing 100176, China; 2 Australian National Insect Collection, CSIRO National Research Collections Australia, Canberra, ACT 2601, Australia; 3 Research School of Biology, Australian National University, Canberra, ACT 2601, Australia

## Abstract

The genus *Dacus* is one of the most economically important tephritid fruit flies. The first complete mitochondrial genome (mitogenome) of *Dacus* species – *D. longicornis* was sequenced by next-generation sequencing in order to develop the mitogenome data for this genus. The circular 16,253 bp mitogenome is the typical set and arrangement of 37 genes present in the ancestral insect. The mitogenome data of D. *longicornis* was compared to all the published homologous sequences of other tephritid species. We discovered the subgenera *Bactrocera*, *Daculus* and *Tetradacus* differed from the subgenus *Zeugodacus*, the genera *Dacus*, *Ceratitis* and *Procecidochares* in the possession of TA instead of TAA stop codon for *COI* gene. There is a possibility that the TA stop codon in *COI* is the synapomorphy in *Bactrocera* group in the genus *Bactrocera* comparing with other Tephritidae species. Phylogenetic analyses based on the mitogenome data from Tephritidae were inferred by Bayesian and Maximum-likelihood methods, strongly supported the sister relationship between *Zeugodacus* and *Dacus*.

The genus *Dacus* Fabricius (Diptera: Tephritidae: Dacini) is one of the most economically important fruit flies^1^. There are about 248 *Dacus* species most of which show a strong preference for attacking the pods of Asclepiadaceae and Apocynaceae, or the fruits and flowers of Cucurbitaceae^1^,^2^. The majority of *Dacus* species distribute in the African continent, several species are found in the Indian Subcontinent, Southeast Asia, Australia and the Pacific^2^.

*Dacus (Callantra) longicornis* Wiedemann has a widespread distribution across southern Asia and Southeast Asia and attacks Cucurbitaceae species^3^. Very limited studies focused on *D. longicornis* are available except for taxonomy and records or first record of this species in some countries and areas^3^,^4^,^5^. Molecular data of *D. longicornis* has not been well studied with only seven records published in GenBank as of May 2016. It is becoming increasingly evident that detailed knowledge of molecular data of *D. longicornis* is required not only for its population structure and geographical variability studies, but also for a comprehensive phylogeny analysis of the tribe Dacini which consist of two very large genera - *Bactrocera* Macquart (629 spp.) and *Dacus* Fabricius (248 spp.) and two small genera - *Ichneumonopsis* Hardy (one sp.) and *Monacrostichus* Bezzi (two spp.)^2^,^7^,^8^,^9^.

The whole mitogenome has become established as one of the most useful markers and has been used for molecular systematic, phylogeography, diagnostics and molecular evolutionary studies^10^,^11^,^12^. By the May of 2016, forty-five complete mitogenomes of 19 Tephritidae species are available in GenBank (Supplementary Table S1), including 16 *Bactrocera* species which are *Bactrocera (Bactrocera) arceae* (Hardy & Adachi) (KR233259)^8^, *B. (B.) carambolae* Drew & Hancock (EF014414), *B. (B.) correcta* (Bezzi) (JX456552), *B. (B.) dorsalis* (Hendel) (DQ845759, DQ917577, *B. (B.) papayae* Drew & Hancock DQ917578 and *B. (B.) philippinensis* Drew & Hancock DQ995281; *B. (B.) papayae* and *B. (B.) philippinensis* have been proven to be the same species with *B. (B.) dorsalis*)^13^,^14^, *B. (B.) latifrons* (Hendel) (KT881556)^9^, *B. (B.) melastomatos* Drew & Hancock (KT881557)^9^, *B. (B.) tryoni* (Froggatt) (HQ130030)^15^, *B. (B.) umbrosa* (Fabricius) (KT881558)^9^, *B. (B.) zonata* (Saunders) (KP296150)^16^, *B. (Daculus) oleae* (Gmelin) (AY210702, AY210703, GU108459 to GU108479)^15^,^17^, *B. (Tetradacus) minax* (Enderlein) (HM776033)^18^, *B. (Zeugodacus) caudata* (Fabricius) (KT625491 and KT625492)^9^, *B. (Z.) cucurbitae* (Coquillett) (JN635562)^19^, *B. (Z.) diaphora* (Hendel) (KT159730)^20^, *B. (Z.) scutellata* (Hendel) (KP722192) and *B. (Z.) tau* (Walker) (KP711431)^21^; one *D. longicornis* (KX345846) (from this study); one *Ceratitis (Ceratitis) capitata* (Wiedemann) (AJ242872)^22^ and one *Procecidochares utilis* Stone (KC355248).

Several studies also did phylogenetic analysis of tephritid species based on the published mitogenome sequences^8^,^9^,^16^, however, sixteen of the 19 published species belong to the genus *Bactrocera*, so that the phylogenetic relationships of higher taxon cannot be explained very well. Recently, the phylogenetic study of Dacini has been attracted more attention especially in the taxonomic status of the subgenus *Zeugodacus*. Some researchers recommended to raising *Zeugodacus* to genus level^6^, and because of the limited molecular data, some researchers also suggested that the exact relationship between *Zeugodacus*, *Dacus* and *Bactrocera* still needs to be properly resolved^7^.

In this study, we report the first complete mitogenome of *Dacus* species - *D. longicornis*, compare the mitogenome data with other tephritid species, and discuss the molecular phylogeny of Dacini in particular.

## Results

### Sequencing and assembly of Mitogenome

An Illumina library was constructed on the DNA of *Dacus longicornis* with an average insert size of 480 bp. This library was sequenced on a run of Hiseq2500 and following removal of sequencing adapters, 9,857,102 read-pairs were generated. Approximately 8% of the reads resembled mitochondrial sequences after BLASTn filtering with E ≤ 1e-5. Assemblies constructed with the IDBA-UD assemblers^23^ resulted in 10 contigs more than 10,000 bp in length of which the longest contig represented complete mitogenome of *D. longicornis*.

### Mitogenome features

**Figure 1:**
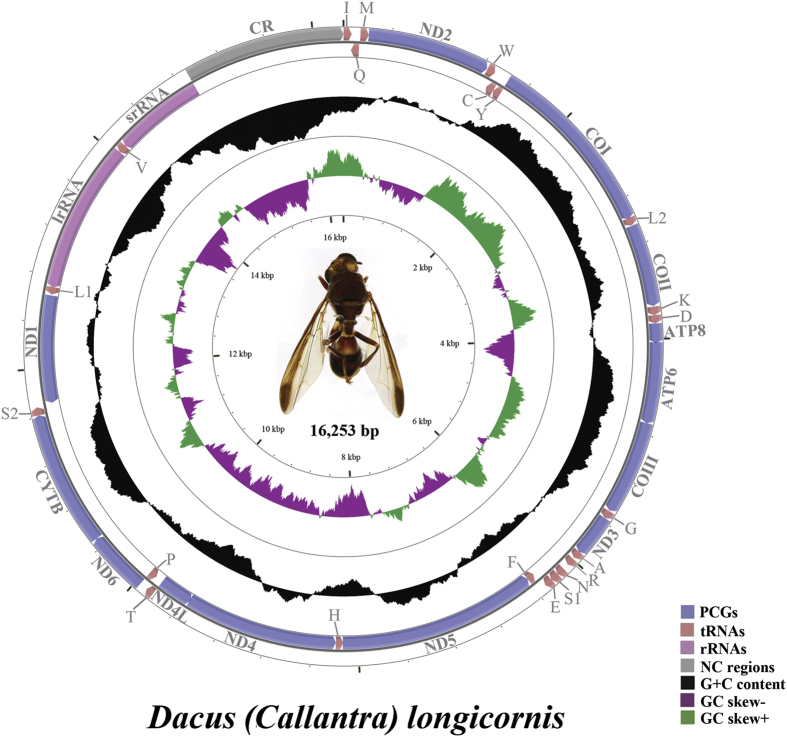
Mitochondrial genome map of *Dacus longicornis*. Arrows indicate the orientation of gene transcription. tRNA genes are indicated with their one-letter corresponding amino acids (L1: CUN; L2: UUR; S1: AGN; S2: UCN). The GC content was plotted using a black sliding window, as the deviation from the average GC content of the entire sequence. GC-skew was plotted as the deviation from the average GC-skew of the entire sequence.

The complete mitogenome of *D. longicornis* was 16,253 bp in length. It presented the typical set and arrangement of 37 genes found in the ancestral insect mitochondrial genome, including 13 protein-coding genes (PCGs), two ribosomal RNA (rRNA) genes, 22 transfer RNA (tRNA) genes and a control region (A + T-rich region) (Table 1; Fig. 1). Nine PCGs (*ND2*, *COI*, *COII*, *COIII*, *ATP6*, *ATP8*, *ND3*, *ND6* and *CYTB*), 14 tRNAs (*tRNA*^*Ile*^, *tRNA*^*Met*^, *tRNA*^*Trp*^, *tRNA*^*Leu(UUR)*^, *tRNA*^*Lys*^, *tRNA*^*Asp*^, *tRNA*^*Gly*^, *tRNA*^*Ala*^, *tRNA*^*Arg*^, *tRNA*^*Asn*^, *tRNA*^*Ser(AGN)*^, *tRNA*^*Glu*^, *tRNA*^*Thr*^ and *tRNA*^*Ser(UCN)*^) and the control region were located on the major strand (J-strand). Four PCGs (*ND5*, *ND4*, *ND4L* and *ND1*), eight tRNAs (*tRNA*^*Gln*^, *tRNA*^*Cys*^, *tRNA*^*Tyr*^, *tRNA*^*Phe*^, *tRNA*^*His*^, *tRNA*^*Pro*^, *tRNA*^*Leu(CUN)*^ and *tRNA*^*Val*^) and two rRNAs (*lrRNA* and *srRNA*) were located on the minor strand (N-strand).

Spacing sequences in 14 regions ranged from 1 to 49 bp, the longest located between *tRNA*^*Arg*^ and *tRNA*^*Asn*^. The overlapping sequences ranged from 1 to 8 bp in 13 regions, the longest was between *tRNA*^*Trp*^ and *tRNA*^*Cys*^.

As in other insect mitogenomes^24^, the nucleotide composition of *D. longicornis* was all AT biased and positive AT skews and negative GC skews, not only in the whole mitochondrial genome but also in PCGs, rRNAs, tRNAs and the control region (Table 2).

All of the PCGs started with ATN codons (ATG in *COII*, *ATP6*, *COIII*, *ND4*, *ND4L*, *CYTB* and *ND1*; ATC in *ATP8*, *ND5* and *ND6*; ATT in *ND2*; ATA in *ND3*) except for *COI* which started with TCG codon. Seven PCGs (*COI*, *COII*, *ATP8*, *ATP6*, *COIII*, *ND4L* and *ND6*) stopped with TAA codon, three PCGs (*ND2*, *ND3* and *ND4*) had TAG stop codon, while *ND5*, *CYTB* and *ND1* had incomplete stop codon T.

**Figure 2:**
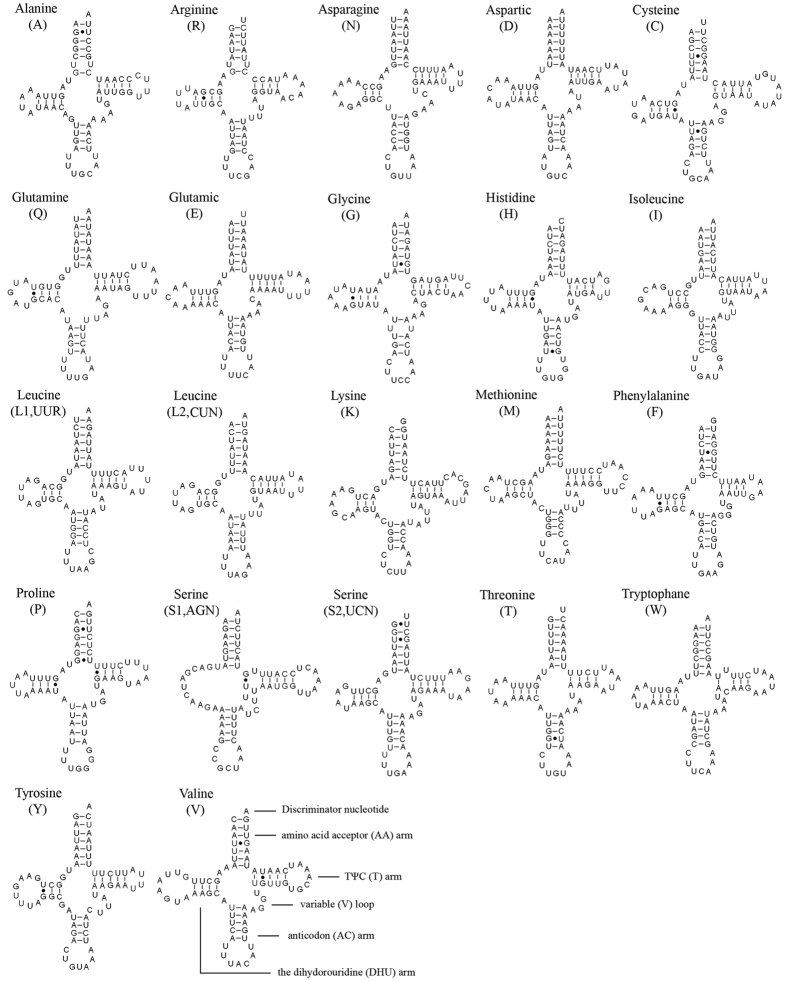
Putative secondary structures of tRNAs found in the mitochondrial genome of *Dacus longicornis*. The tRNAs are labelled with the abbreviations of their corresponding amino acids. Inferred Watson-Crick bonds are illustrated by lines, whereas GU bonds are illustrated by dots.

Twenty-two typical tRNAs which are usually observed in insect mitogenomes were also found in *D. longicornis* mitogenome. The size of 22 tRNAs ranged from 64 bp (*tRNA*^*His*^) to 72 bp (*tRNA*^*Val*^). Most tRNAs could be folded into the cloverleaf structure except for *tRNA*^*Ser(AGN)*^ which lacked the dihydorouridine (DHU) arm (Fig. 2). Twenty-three G-U pairs, four mismatched base U-U pairs and one mismatched base U-C pair were found in *D. longicornis* mitogenome tRNA secondary structures. The G-U pairs were located in the amino acid acceptor (AA) arm (9 bp), DHU arm (8 bp), anticodon (AC) arm (3 bp) and TψC (T) arm (3 bp). The mismatched base U-U pairs were located in AA arm (2 bp), AC arm (1 bp) and T arm (1 bp). The mismatched base U-C pairs were located in T arm.

The *lrRNA* was assumed to fill up the blanks between *tRNA*^*Leu(CUN)*^ and *tRNA*^*Val*^. For the boundary between the *srRNA* gene and the control region, alignments with homologous sequences in other mitogenomes of Tephritidae were applied to determine the 3′-end of the gene. The *lrRNA* is 1,331 bp long with an A + T content of 78.5%, and the *srRNA* is 798 bp long with an A + T content of 74.9%.

The control region (1,343 bp) was flanked by *srRNA* and *tRNA*^*Ile*^ and was highly enriched in AT (85.3%). Two 151 bp repeats were found in the control region and one 19 bp poly-T stretch located near the repeats. Furthermore, the region near *tRNA*^*Ile*^ contained another 22 bp poly-A stretch. Both repeated sequences and poly stretches are common in the control region for most insects^25^,^26^, and these motifs may function during processing of the replication and transcription.

### Phylogenetic relationship

Four datasets were used in the phylogenetic analysis, there are 14,586 residues in the PCG123RNA matrix (containing nucleotides of 13 PCGs, two rRNAs and 22 tRNAs), 11,148 residues in the PCG123 matrix (containing nucleotides of 13 PCGs), 10,870 residues in the PCG12RNA matrix (containing nucleotides of 13 PCGs but excluding the third codon sites, two rRNAs and 22 tRNAs) and 7,432 residues in the PCG12 matrix (containing nucleotides of 13 PCGs but excluding the third codon sites).

**Figure 3:**
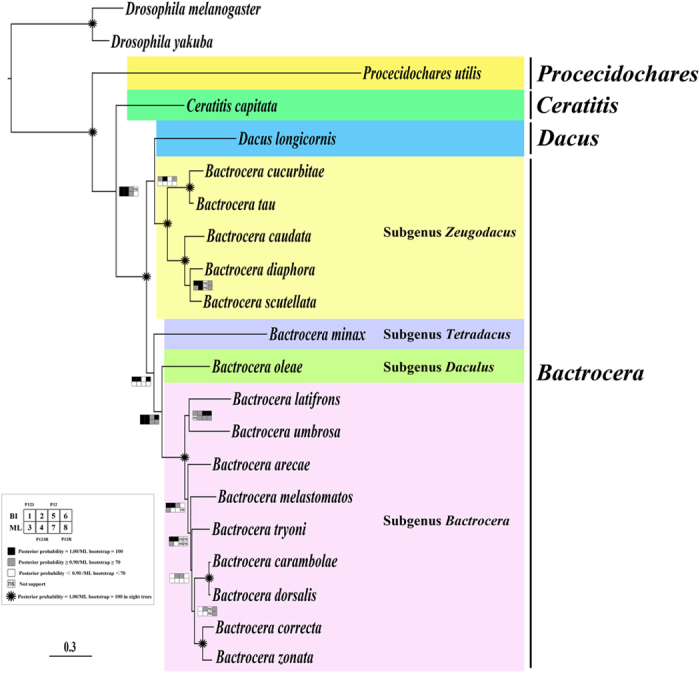
Phylogenetic tree of Tephritidae family based on mitochondrial genomes. Squares at the nodes are Bayesian posterior probabilities for 1, 2, 5 and 6, ML bootstrap values for 3, 4, 7 and 8. Dataset of PCG123, 1 and 3, PCG123RNA, 2 and 4, PCG12, 5 and 7, PCG12RNA, 6 and 8. Black indicates posterior probabilities = 1.00 or ML bootstrap = 100, gray indicates posterior probabilities ≥ 0.90 or ML bootstrap ≥ 70, white indicates posterior probabilities <0.90 or ML bootstrap < 70, ‘ns’ indicates not support, *indicates posterior probabilities = 1.00 or ML bootstrap = 100 in eight trees.

The topology structures conducted from Bayesian and ML analyses were very similar based on these four datasets (Fig. 3). The monophyly of Tephritidae and Dacini tribe were well supported in all trees with posterior probabilities 1.0 and ML bootstraps 100. The genus *Bactrocera* was not monophyletic but it was different from other Tephritidae mitochondrial genome phylogeny studies which only included *Bactrocera* speices of Dacini^8^,^9^,^16^,^20^,^21^. Members of the subgenera *Bactrocera* and *Zeugodacus* formed a distinct clade from the other subgenera *Daculus* and *Tetradacus*, respectively. The subgenus *Zeugodacus* and *Callantra* were sister groups from our results, which supported the conclusions from several recent studies about phylogenetic relationship of Dacini^6^,^7^.

## Discussion

In this study, we are reporting the first complete mitochondrial genome of *Dacus* species - *Dacus longicornis* in Dacini tribe of Tephritidae. The size of *D. longicornis* mitogenome is 16,253 bp, which is the largest one among the other 18 tephritid mitogenomes available with the size ranging from 15,687 bp in *B. tau* to 16,043 bp in *B. minax*. The control region of *D. longicornis* mitogenome is 1,343 bp in length, which is also the longest one in the other published tephritid mitogenomes with the size ranging from 801 bp in *B. tau* to 1,141 bp in *B. minax* (Supplementary Table S2).

The A + T contents of the whole mitogenome, PCGs, tRNAs, rRNAs and CR in *D. longicornis* are 72.33%, 69.4%, 74.81%, 77.17% and 85.26%, average amongst all reported tephritid mitogenomes, which range from 67.28% (*B. minax*) to 80.83% (*P. utilis*) in the whole mitogenome, from 64.30% (*B. minax*) to 78.90% (*P. utilis*) in PCGs, from 72.31% (*B. minax*) to 80.61% (*P. utilis*) in tRNAs, from 73.71% (*B. minax*) to 85.69% (*P. utilis*) in rRNAs and from 77.65% (*B. minax*) to 91.14% (*C. capitata*) in CR (Supplementary Table S2).

The AT skews and GC skews of *D. longicornis* in the whole mitogenome, PCGs, tRNAs, rRNAs and CR are 0.101 (from 0.021 in *C. capitata* to 0.131 in *B. minax*) and −0.293 (from −0.175 in *P. utilis* to −0.316 in *B. minax*), 0.105 (from 0.019 in *C. capitata* to 0.148 in *B. minax*) and −0.301 (from −0.170 in *P. utilis* to −0.319 in *B. minax*), 0.052 (from 0.005 in *P. utilis* to 0.055 in *B. minax*) and −0.126 (from −0.074 in *B. cucurbitae* to −0.182 in *B. minax*), 0.087 (from 0.051 in *P. utilis* to 0.121 in *B. minax*) and −0.329 (from −0.267 in *C. capitata* to −0.356 in *B. minax*), 0.146 (maximum) and −0.354 (minimum), respectively. The CR of *D. longicornis* shows the most marked AT skews and GC skews compared with the other tephritid mitogenomes (Supplementary Table S2).

Similar to the other tephritid mitogenomes, tRNAs of *D. longicornis* have three main clusters: (1) *tRNA*^*Ile*^ – *tRNA*^*Gln*^ – *tRNA*^*Met*^; (2) tRNA^Trp^ – tRNA^Cys^ – tRNA^Tyr^; (3) *tRNA*^*Ala*^ – *tRNA*^*Arg*^ – *tRNA*^*Asn*^ – *tRNA*^*Ser(AGN)*^ – *tRNA*^*Glu*^ – *tRNA*^*Phe*^. The atypical cloverleaf structure of *tRNA*^*Ser(AGN)*^ is similar to this gene in other metazoan mitogenomes^27^.

Seven PCGs in all Tephritidae species have the same start codons (ATG in *ATP6*, *COII*, *CYTB*, *ND4* and *ND4L*, ATT in *ND2*, TCG in *COI*), and five PCGs (*ATP6*, *ATP8*, *COIII*, *ND4L* and *ND6*) have the same stop TAA codons (Table 3). It’s worth noting that the subgenera *Bactrocera*, *Daculus* and *Tetradacus* differ from the subgenus *Zeugodacus*, the genera *Dacus*, *Ceratitis* and *Procecidochares* in the possession of TA instead of TAA stop codon for *COI* gene. There is a possibility that the TA stop codon in COI is the synapomorphy in *Bactrocera* group which includes 10 subgenera (*Afrodacus*, *Apodacus*, *Bactrocera*, *Bulladacus*, *Daculus*, *Gymnodacus*, *Notodacus*, *Semicallantra*, *Tetradacus* and *Trypetidacus*) in the genus *Bactrocera*^28^,^29^ comparing with other Tephritidae species.

Studies on molecular phylogenetic relationship of Dacini fruit flies have been reported by several researchers. Early study supported that *Bactrocera* and *Dacus* were each monophyletic based on phylogenetic analysis of 34 tephritid fruit flies including 16 species of Dacini, utilizing 1,391 bp from *COII*, *lrRNA*, *srRNA*, *tRNA*^*Lys*^ and *tRNA*^*Asp*^ genes^30^. Four years later, Segura *et al*. reported that *B. cucurbitae* was more closely related to the genus *Dacus* than to other *Bactrocera* species using *CYTB*, *ND1* and *tRNA*^*Ser*^ genes from 23 tephritid species, and White also suggested that the subgenus *Zeugodacus* might be a sister group to the genus *Dacus* in the same year^31^,^32^. In recent four years, Krosch *et al*. concluded that ‘Zeugodacus’ clade was the sister group to *Dacus* based on *COI*, *COII*, *lrRNA* and white eye genes from 125 Dacini species^6^. Virgilio *et al*. also drew the same conclusion according to two datasets. One dataset included 98 vouchers using *COI*, *ND6*, *lrRNA*, *tRNA*^*Pro*^ and period genes and the other included 159 vouchers based on *COI* and *lrRNA* genes^7^. Here we added support for this conclusion that *Zeugodacus* and *Dacus* are sister groups from mitochondrial genome data.

As for the taxonomic status of the subgenus *Zeugodacus*, Krosch *et al*. suggested that taxonomic consideration should be given to raising Zeugodacus to genus level^6^. Virgilio *et al*. supported this conclusion, but also proposed that the exact relationship between *Zeugodacus*, *Dacus* and *Bactrocera* still needed to be properly resolved^7^. Considering that there are 30 recognized subgenera four groups in the genus *Bactrocera* and eight subgenera in *Dacus*^33^, and the subgenera *Bactrocera* and *Zeugodacus* have been proven to be not monophyletic^34^,^35^, we suggest to accurately resolve the exact relationship of Dacini with more complete taxon sampling and more comprehensive molecular data combining mitochondrial genomes and nuclear genes. Raising *Zeugodacus* to genus level is also needed to be confirmed further based on more taxonomy, biology and biogeography evidence.

## Materials and Methods

### Sample collection

The adult specimen of *D. longicornis* was collected from Yunnan province of China. It was identified based on available taxonomic keys^3^, and preserved in absolute ethyl alcohol and stored in −20 °C freezer in Chinese Academy of Inspection and Quarantine until use.

### Mitogenome sequencing and analysis

The genomic DNA was extracted from one fly’s muscle tissues of the thorax using the DNeasy DNA Extraction kit (QIAGEN) following the manufacturer’s instructions. The concentration of double-stranded DNA (dsDNA) in extraction was assayed on a Qubit fluorometer using a dsDNA high-sensitivity kit (Invitrogen).

An Illumina TruSeq library was generated from the genomic DNA with an average insert size of 480 bp. The library was sequenced on a full run of Illumina Hiseq2500 with 500 cycles and paired-end sequencing (250 bp reads).

A quality assessment of raw FASTQ files for the library was made using FastQCv0.11.4 (www.bioinformatics.babraham.ac.uk/projects/fastqc) prior to the removal of adapter sequences with Trimmomatic v0.30 (ILLUMINACLIP:2:30:10)^36^. The putative mitochondrial reads were identified in a BLASTn search^37^ against a custom reference of Tephritidae mitogenomes (E ≤ 1e-5; maximum target sequences 1; DUST filtering disabled). The extracted mitochondrial reads were subjected to whole-genome shot-gun assembly using IDBA-UD^23^. Assemblies with IDBA-UD used a similarity threshold of 98% and minimum and maximum k values of 80 and 240 bp, respectively. Following assembly, the contig identified as mitogenome was manually checked in Geneious (http://www.geneious.com/) for identical or near-identical overlapping terminal regions and were circularized where possible.

Protein-coding genes (PCGs) and two ribosomal RNA (rRNA) genes were identified by BLAST searches in NCBI (http://www.ncbi.nlm.nih.gov/) and confirmed by alignment with homologous genes from other 18 tephritid species available in GenBank. Transfer RNA (tRNA) genes were identified using the tRNAscan-SE^38^ and ARWEN^39^ and checked manually. The circular map of *D. longicornis* mitogenome sequence was drawn with CGView^40^. The nucleotide composition and codon usage were analyzed using MEGA 6.0^41^. The composition of skew was measured with the following formula: AT skew = (A − T)/(A + T) and GC skew = (G − C)/(G + C)^42^. The annotated mitogenome sequence of *D. longicornis* has been deposited in GenBank with accession number KX345846.

### Phylogenetic analyses

To better resolve molecular phylogeny of Dacini especially between *Dacus* and *Zeugodacus*, a total of 21 species of Diptera species were used in phylogenetic analysis, including 19 Tephritidae and two outgroup species from Drosophilidae. Detailed information of these species used in this study were listed in Supplementary Table S1.

Sequences of 13 PCGs, two rRNAs and 22 tRNAs were used in phylogenetic analysis. The MAFFT algorithm in the TranslatorX online platform^43^ under the L-INS-i strategy was utilized to align 13 PCGs based on codon-based multiple alignments and to toggle back to the nucleotide sequences. Before back-translate to nucleotides, poorly aligned sites were removed from the protein alignment using GBlocks within the TranslatorX with default settings. Muscle algorithm implemented in MEGA 6.0^41^ was performed to align the sequences of two rRNAs, ambiguous positions in the rRNAs alignment were filtered by hand. Quality control of the hand alignments^44^ was performed by comparing with homologous sequences from previously sequenced tephritid mitogenomes to identify 22 tRNAs. Individual genes were concatenated using SequenceMatrix v1.7.8^45^. Four datasets were set up for phylogenetic analysis: (1) nucleotides of 13 PCGs, two rRNAs and 22 tRNAs (P123R) with 14,586 residues, (2) nucleotides of 13 PCGs (P123) with 11,148 residues, (3) nucleotides of 13 PCGs exclude the third codon sites, two rRNAs and 22 tRNAs (P12R) with 10,870 residues and (4) nucleotides of 13 PCGs exclude the third codon sites (P12) with 7,432 residues.

The optimal partition strategy and substitution models for each partition were selected by PartitionFinder v1.1.1^46^. As the software required a user to pre-define partitions, we created input configuration files with 39/42/26/29 (P123/P123R/P12/P12R) pre-defined partitions of the dataset. The “greedy” algorithm were used along with branch lengths estimated as “unlinked” and Bayesian information criterion (BIC)^47^,^48^ to search for the best-fit scheme. The best selected partitioning schemes and models of three datasets for ML and BI analyses were listed in Supplementary Table S3.

We performed Bayesian inference (BI) and maximum likelihood (ML) based on the best-fit partitioning schemes recommended by PartitionFinder (Supplementary Table S3). We used MrBayes 3.2.2^49^ to conduct Bayesian analysis. The datasets were conducted with two simultaneous runs of 2 million generations, each with one cold and three heated chains. Samples were drawn every 1,000 Markov chain Monte Carlo (MCMC) steps, with the first 25% discarded as burn-in. The stationarity was considered to be reached and stopped run when the average standard deviation of split frequencies was below 0.01. The ML analysis was conducted with RAxML 8.0.0^50^ with 1,000 bootstrap replicates and using the rapid bootstrap feature (random seed value 12345)^51^.

## Additional Information

**Accession Codes**: Dacus longicornis mitochondrial genome is available in GenBank database (accession number: KX345846).

**How to cite this article**: Jiang, F. *et al*. The first complete mitochondrial genome of *Dacus longicornis* (Diptera: Tephritidae) using next-generation sequencing and mitochondrial genome phylogeny of Dacini tribe. *Sci. Rep.* **6**, 36426; doi: 10.1038/srep36426 (2016).

**Publisher’s note:** Springer Nature remains neutral with regard to jurisdictional claims in published maps and institutional affiliations.

## Supplementary Material

Supplementary Information

## Figures and Tables

**Table 1:** Characteristics of the mitochondrial genome of *Dacus longicornis*.

Gene	Strand	Location	Size (bp)	Anticodon	Codon		Intergenic Sequence
					Start	Stop	
*tRNA*^*Ile*^	J	1–67	67	GAT			
*tRNA*^*Gln*^	N	65–133	69	TTG			−3
*tRNA*^*Met*^	J	141–209	69	CAT			7
*ND2*	J	210–1232	1023		ATT	TAG	0
*tRNA*^*Trp*^	J	1239–1306	68	TCA			6
*tRNA*^*Cys*^	N	1299–1367	69	GCA			−8
*tRNA*^*Tyr*^	N	1369–1435	67	GTA			1
*COI*	J	1434–2972	1539		TCG	TAA	−2
*tRNA*^*Leu (UUR)*^	J	2968–3033	66	TAA			−5
*COII*	J	3038–3727	690		ATG	TAA	4
*tRNA*^*Lys*^	J	3733–3803	71	CTT			5
*tRNA*^*Asp*^	J	3804–3872	69	GTC			0
*ATP8*	J	3873–4034	162		ATC	TAA	0
*ATP6*	J	4028–4705	678		ATG	TAA	−7
*COIII*	J	4705–5493	789		ATG	TAA	−1
*tRNA*^*Gly*^	J	5500–5566	67	TCC			6
*ND3*	J	5567–5920	354		ATA	TAG	0
*tRNA*^*Ala*^	J	5919–5983	65	TGC			−2
*tRNA*^*Arg*^	J	5996–6060	65	TCG			12
*tRNA*^*Asn*^	J	6110–6176	67	GTT			49
*tRNA*^*Ser (AGN)*^	J	6176–6245	70	GCT			−1
*tRNA*^*Glu*^	J	6245–6312	68	TTC			−1
*tRNA*^*Phe*^	N	6331–6395	65	GAA			18
*ND5*	N	6396–8133	1738		ATC	T-	−3
*tRNA*^*His*^	N	8131–8194	64	GTG			15
*ND4*	N	8195–9535	1341		ATG	TAG	0
*ND4L*	N	9529–9825	297		ATG	TAA	−7
*tRNA*^*Thr*^	J	9827–9891	65	TGT			1
*tRNA*^*Pro*^	N	9892–9957	66	TGG			0
*ND6*	J	9960–10481	522		ATC	TAA	2
*CYTB*	J	10481–11615	1135		ATG	T-	−1
*tRNA*^*Ser (UCN)*^	J	11616–11682	67	TGA			0
*ND1*	N	11698–12637	940		ATG	T-	15
*tRNA*^*Leu (CUN)*^	N	12648–12712	65	TAG			10
*lrRNA*	N	12710–14040	1331				−3
*tRNA*^*Val*^	N	14041–14112	72	TAC			0
*srRNA*	N	14113–14910	798				0
A + T rich-region	J	14911–16253	1343				0

**Table 2:** Nucleotide composition of the mitochondrial genome of *Dacus longicornis*.

Region	A%	C%	G%	T%	A + T%	G + C%	AT skew	GC skew
Whole mtDNA	39.8	17.9	9.8	32.5	72.3	27.7	0.101	−0.293
PCGs	38.3	19.9	10.7	31.1	69.4	30.6	0.105	−0.301
tRNAs	39.4	14.2	11.0	35.4	74.8	25.2	0.052	−0.126
rRNAs	41.9	15.2	7.7	35.2	77.2	22.8	0.087	−0.330
CR	48.8	10.0	4.8	36.4	85.3	14.7	0.146	−0.354

**Table 3:** Usage of start and stop codons in mitochondrial genome of Tephritidae.

Species	*ATP6*		*ATP8*		*COI*		*COII*		*COIII*		*CYTB*		*ND1*		*ND2*		*ND3*		*ND4*		*ND4L*		*ND5*		*ND6*	
	start	stop	start	stop	start	stop	start	stop	start	stop	start	stop	start	stop	start	stop	start	stop	start	stop	start	stop	start	stop	start	stop
*D. (C.) longicornis*	ATG	TAA	ATC	TAA	TCG	TAA	ATG	TAA	ATG	TAA	ATG	T	ATG	T	ATT	TAG	ATA	TAG	ATG	TAG	ATG	TAA	ATT	T	ATC	TAA
*B. (B.) arecae*	ATG	TAA	GTG	TAA	TCG	TA	ATG	TAA	ATG	TAA	ATG	TAA	ATA	T	ATT	TAA	ATT	T	ATG	TAG	ATG	TAA	ATC	T	ATT	TAA
*B. (B.) carambolae*	ATG	TAA	GTG	TAA	TCG	TA	ATG	TAA	ATG	TAA	ATG	T	ATA	T	ATT	TAA	ATT	TAG	ATG	TAG	ATG	TAA	ATT	T	ATT	TAA
*B. (B.) correcta*	ATG	TAA	GTG	TAA	TCG	TA	ATG	TAA	ATG	TAA	ATG	TAG	ATA	T	ATT	TAA	ATT	TAG	ATG	TAG	ATG	TAA	ATT	T	ATT	TAA
*B. (B.) dorsalis*	ATG	TAA	GTG	TAA	TCG	TA	ATG	TAA	ATG	TAA	ATG	TAG	ATA	T	ATT	TAA	ATT	T	ATG	TAG	ATG	TAA	ATT	T	ATT	TAA
*B. (B.) latifrons*	ATG	TAA	GTG	TAA	TCG	TA	ATG	TAA	ATG	TAA	ATG	TAA	ATA	T	ATT	TAA	ATT	T	ATG	TAG	ATG	TAA	ATT	T	ATT	TAA
*B. (B.) melastomatos*	ATG	TAA	GTG	TAA	TCG	TA	ATG	TAA	ATG	TAA	ATG	T	ATA	T	ATT	TAA	ATC	T	ATG	TAG	ATG	TAA	ATT	T	ATC	TAA
*B. (B.) tryoni*	ATG	TAA	GTG	TAA	TCG	TA	ATG	TAA	ATG	TAA	ATG	TAG	ATA	T	ATT	TAA	ATT	T	ATG	TAG	ATG	TAA	ATT	T	ATT	TAA
*B. (B.) umbrosa*	ATG	TAA	ATG	TAA	TCG	TA	ATG	TAA	ATG	TAA	ATG	T	ATA	T	ATT	TAA	ATT	T	ATG	TAG	ATG	TAA	ATT	T	ATT	TAA
*B. (B.) zonata*	ATG	TAA	GTG	TAA	TCG	TA	ATG	TAA	ATG	TAA	ATG	TAG	ATA	T	ATT	TAA	ATT	T	ATG	TAG	ATG	TAA	ATT	T	ATT	TAA
*B. (D.) oleae*	ATG	TAA	ATC	TAA	TCG	TA	ATG	TAA	ATG	TAA	ATG	TAG	ATG	T	ATT	TAA	ATC	TAG	ATG	TAA	ATG	TAA	ATT	TAA	ATC	TAA
*B. (T.) minax*	ATG	TAA	ATT	TAA	TCG	TA	ATG	TAA	ATG	TAA	ATG	TAG	ATA	T	ATT	TAG	ATC	T	ATG	TAA	ATG	TAA	ATT	TAA	ATG	TAA
*B. (Z.) caudata*	ATG	TAA	ATT	TAA	TCG	TAA	ATG	TAA	ATG	TAA	ATG	T	ATA	T	ATT	TAA	ATC	TAG	ATG	TAA	ATG	TAA	ATT	T	ATT	TAA
*B. (Z.) cucurbitae*	ATG	TAA	ATT	TAA	TCG	TAA	ATG	TAA	ATG	TAA	ATG	T	ATA	T	ATT	TAA	ATC	TAG	ATG	TAA	ATG	TAA	ATT	T	ATT	TAA
*B. (Z.) diaphora*	ATG	TAA	ATT	TAA	TCG	TAA	ATG	TAA	ATG	TAA	ATG	TAG	ATA	T	ATT	TAA	ATC	T	ATG	TAA	ATG	TAA	ATT	T	ATT	TAA
*B. (Z.) scutellata*	ATG	TAA	ATT	TAA	TCG	TAA	ATG	TAA	ATG	TAA	ATG	TAG	ATA	T	ATT	TAA	ATC	TAG	ATG	TAA	ATG	TAA	ATT	T	ATT	TAA
*B. (Z.) tau*	ATG	TAA	ATT	TAA	TCG	TAA	ATG	TAA	ATG	TAA	ATG	TAG	ATA	T	ATT	TAA	ATC	TAA	ATG	TAA	ATG	TAA	ATT	T	ATT	TAA
*C. capitata*	ATG	TAA	ATT	TAA	TCG	TAA	ATG	TAA	ATG	TAA	ATG	T	ATT	T	ATT	TAA	ATA	TAA	ATG	TAA	ATG	TAA	ATT	T	ATT	TAA
*P. utilis*	ATG	TAA	ATT	TAA	TCG	TAA	ATG	T	ATA	TAA	ATG	TAA	ATA	TAG	ATT	TAA	ATT	TAA	ATG	TAA	ATG	TAA	ATT	T	ATA	TAA
