# Supplementary material for: The first complete mitochondrial genome of Dacus longicornis (Diptera: Tephritidae) using next-generation sequencing and mitochondrial genome phylogeny of Dacini tribe
Source: Sci Rep. 2016 Nov 4;6:36426. doi: 10.1038/srep36426 (PMC5095552; doi:10.1038/srep36426)
Supplement: Supplementary Information [file srep36426-s1.pdf]

The first complete mitochondrial genome of *Dacus longicornis* (Diptera: Tephritidae) using next-generation sequencing and mitochondrial genome phylogeny of Dacini tribe

Fan Jiang[1], Xubin Pan[1], Xuankun Li[2,3], Yanxue Yu[1], Junhua Zhang[1], Hongshan Jiang[1], Liduo Dou[1], Shuifang Zhu[1*]

[1]Institute of Plant Quarantine, Chinese Academy of Inspection and Quarantine, Beijing 100176, China.

[2]Australian National Insect Collection, CSIRO National Research Collections Australian, Canberra, ACT 2601, Australia.

[3]Research School of Biology, Australian National University, Canberra, ACT 2601, Australia.

[*] Corresponding author: zhusf@caiq.gov.cn

Table S1. Mitochondrial genome of Tephritidae in GenBank (by the end of May, 2016).

Genus	Subgenus	Species	Accession Number	Population
Bactrocera	*Bactrocera*	*B. arecae*	KR233259	Kuala Lumpur, Malaysia
		B. carambolae	EF014414	Japan
		B. correcta	JX456552	Yunnan, China
		B. dorsalis	DQ845759	Guangdong, China
			DQ917577	Guangdong, China
			DQ917578	Malaysia
			DQ995281	Philippines
		B. latifrons	KT881556	Malaysia
		B. melastomatos	KT881557	Malaysia
		B. tryoni	HQ130030	/
		B. umbrosa	KT881558	Malaysia
		B. zonata	KP296150	Ranchi, India
	Daculus	*B. oleae*	AY210702	Mirandela, Portugal
			AY210703	Siena, Italy
			GU108459	Italy
			GU108460	Israel
			GU108461	Turkey
			GU108462	South Africa
			GU108463	Pakistan
			GU108464	Italy

		GU108465	Turkey
		GU108466	Kenya
		GU108467	Pakistan
		GU108468	Algeria
		GU108469	Pakistan
		GU108470	Italy
		GU108471	Italy
		GU108472	Israel
		GU108473	Portugal
		GU108474	Morocco
		GU108475	USA
		GU108476	Kenya
		GU108477	Pakistan
		GU108478	South Africa
		GU108479	USA
Tetradacus	*B. minax*	HM776033	Chongqing, China
Zeugodacus	*B. caudata*	KT625491	Malaysia
		KT625492	Indonesia
	B. cucurbitae	JN635562	Yunnan, China
	B. diaphora	KT159730	Chongqing, China
	B. scutellata	KP722192	Guangdong, China
	B. tau	KP711431	Guangdong, China

Ceratitis	*Ceratitis*	*C. capitata*	AJ242872	Laboratory Strain
Dacus	*Callantra*	*D. longicornis*	KX345846	Yunnan, China
Procecidochares		*P. utilis*	KC355248	Yunnan, China

Gray indicates the mitochondrial genome data used in the phylogenetic analysis.

Table S2. Nucleotide composition of the mitochondrial genome of *Dacus longicornis*.

Species	GenBank Number	Whole mtDNA				PCGs				tRNAs				rRNAs				CR			
		length	(A+T) %	AT skew	GC skew	length	(A+T) %	AT skew	GC skew	length	(A+T) %	AT skew	GC skew	length	(A+T) %	AT skew	GC skew	length	(A+T) %	AT skew	GC skew
B. arecae	KR233259	15900	72.26	0.081	-0.259	11182	69.66	0.086	-0.265	1467	74.47	0.035	-0.131	2111	77.12	0.095	-0.329	952	86.03	0.060	-0.128
B. carambolae	EF014414	15915	73.55	0.066	-0.224	11192	71.10	0.072	-0.228	1466	75.10	0.019	-0.096	2113	77.57	0.079	-0.304	950	87.89	0.049	-0.130
B. correcta	JX456552	15936	73.17	0.063	-0.222	11192	71.22	0.070	-0.221	1470	75.31	0.024	-0.113	2117	77.85	0.080	-0.322	949	78.61	0.019	-0.163
B. dorsalis	DQ845759	15915	73.58	0.068	-0.228	11185	71.12	0.073	-0.235	1467	75.19	0.017	-0.093	2123	77.81	0.076	-0.287	949	88.09	0.062	-0.186
	DQ917578	15915	73.52	0.066	-0.226	11190	71.04	0.073	-0.234	1466	75.38	0.024	-0.086	2114	77.72	0.069	-0.287	950	88.21	0.064	-0.161
	DQ995281	15915	73.63	0.066	-0.224	11192	71.18	0.071	-0.229	1466	75.31	0.020	-0.099	2114	77.67	0.074	-0.292	949	88.20	0.061	-0.179
B. latifrons	KT881556	15977	71.11	0.088	-0.266	11184	68.10	0.099	-0.275	1466	73.47	0.042	-0.136	2120	77.22	0.084	-0.317	953	86.78	0.069	-0.238
B. melastomatos	KT881557	15954	73.79	0.073	-0.251	11187	71.23	0.078	-0.254	1468	75.48	0.029	-0.133	2114	78.10	0.087	-0.305	953	88.98	0.059	-0.257
B. tryoni	HQ130030	15925	72.42	0.070	-0.227	11187	69.61	0.074	-0.236	1467	74.98	0.022	-0.090	2115	77.73	0.073	-0.295	951	86.96	0.083	-0.129

B. umbrosa	KT881558	15898	70.49	0.084	-0.244	11187	67.27	0.090	-0.245	1465	74.13	0.042	-0.145	2120	77.03	0.094	-0.343	944	86.23	0.049	-0.046
B. zonata	KP296150	15935	73.34	0.065	-0.223	11190	70.99	0.070	-0.224	1469	74.95	0.023	-0.103	2120	78.07	0.077	-0.308	950	84.42	0.040	-0.216
B. oleae	AY210702	15815	72.63	0.088	-0.280	11189	70.18	0.091	-0.288	1466	74.76	0.040	-0.130	2116	77.13	0.099	-0.347	949	86.93	0.091	-0.177
	AY210703	15815	72.59	0.088	-0.280	11189	70.15	0.091	-0.287	1466	74.69	0.041	-0.132	2116	77.08	0.099	-0.344	949	86.83	0.090	-0.184
	GU108459	15814	72.60	0.088	-0.281	11184	70.18	0.092	-0.289	1466	74.76	0.040	-0.130	2116	77.08	0.099	-0.344	948	86.71	0.083	-0.190
	GU108460	15817	72.63	0.089	-0.282	11184	70.21	0.092	-0.291	1466	74.69	0.039	-0.127	2116	77.08	0.100	-0.348	951	86.86	0.087	-0.184
	GU108461	15816	72.65	0.089	-0.282	11184	70.22	0.092	-0.291	1466	74.76	0.040	-0.130	2116	77.08	0.100	-0.344	950	86.95	0.090	-0.194
	GU108462	15814	72.63	0.089	-0.281	11184	70.23	0.091	-0.290	1466	74.69	0.039	-0.127	2115	77.07	0.101	-0.348	949	86.72	0.089	-0.190
	GU108463	15821	72.52	0.089	-0.281	11184	70.07	0.093	-0.290	1466	74.56	0.038	-0.126	2116	76.89	0.100	-0.350	949	87.04	0.085	-0.187
	GU108464	15814	72.59	0.089	-0.281	11184	70.16	0.091	-0.290	1466	74.76	0.040	-0.130	2116	77.08	0.099	-0.344	948	86.71	0.088	-0.190
	GU108465	15816	72.65	0.088	-0.280	11184	70.23	0.091	-0.287	1467	74.64	0.039	-0.124	2115	77.07	0.099	-0.348	950	86.95	0.087	-0.194
	GU108466	15813	72.63	0.089	-0.281	11184	70.23	0.091	-0.290	1466	74.69	0.039	-0.127	2115	77.07	0.101	-0.348	948	86.71	0.090	-0.190
	GU108467	15821	72.52	0.089	-0.281	11184	70.07	0.093	-0.290	1466	74.56	0.038	-0.126	2116	76.89	0.100	-0.350	949	87.04	0.085	-0.187

	GU108468	15815	72.61	0.088	-0.279	11184	70.19	0.091	-0.289	1466	74.69	0.039	-0.127	2116	77.08	0.099	-0.344	949	86.83	0.090	-0.184
	GU108469	15815	72.53	0.089	-0.281	11184	70.09	0.093	-0.290	1467	74.64	0.039	-0.124	2115	76.83	0.100	-0.351	949	87.04	0.085	-0.187
	GU108470	15815	72.61	0.089	-0.281	11184	70.19	0.091	-0.290	1466	74.76	0.040	-0.130	2116	77.08	0.099	-0.344	949	86.72	0.089	-0.190
	GU108471	15813	72.60	0.089	-0.280	11184	70.16	0.092	-0.289	1466	74.76	0.040	-0.130	2116	77.08	0.099	-0.344	947	86.80	0.088	-0.184
	GU108472	15814	72.66	0.088	-0.280	11184	70.25	0.091	-0.289	1467	74.64	0.039	-0.124	2115	77.07	0.099	-0.348	948	86.92	0.085	-0.194
	GU108473	15815	72.61	0.089	-0.280	11184	70.20	0.091	-0.289	1466	74.69	0.039	-0.127	2116	77.08	0.099	-0.344	949	86.72	0.094	-0.175
	GU108474	15817	72.61	0.089	-0.279	11184	70.15	0.091	-0.288	1466	74.76	0.040	-0.130	2116	77.08	0.100	-0.348	951	86.96	0.091	-0.177
	GU108475	15819	72.67	0.089	-0.283	11184	70.23	0.093	-0.293	1466	74.69	0.039	-0.127	2116	77.13	0.099	-0.347	949	86.93	0.088	-0.177
	GU108476	15816	72.62	0.088	-0.279	11184	70.22	0.091	-0.287	1466	74.69	0.039	-0.127	2116	76.98	0.101	-0.347	950	86.84	0.091	-0.200
	GU108477	15815	72.52	0.089	-0.281	11184	70.07	0.093	-0.290	1466	74.62	0.038	-0.124	2115	76.83	0.100	-0.351	950	87.16	0.087	-0.197
	GU108478	15815	72.64	0.089	-0.282	11184	70.23	0.092	-0.290	1466	74.69	0.039	-0.127	2115	77.07	0.101	-0.348	950	86.74	0.090	-0.190
	GU108479	15820	72.67	0.090	-0.283	11184	70.23	0.093	-0.293	1466	74.69	0.039	-0.127	2116	77.13	0.099	-0.347	950	86.95	0.090	-0.177
B. minax	HM776033	16043	67.28	0.131	-0.316	11183	64.30	0.148	-0.319	1466	72.31	0.055	-0.182	2115	73.71	0.121	-0.356	1141	77.65	0.081	-0.333

B. caudata	KT625491	15866	73.15	0.074	-0.252	11193	71.01	0.083	-0.260	1472	74.60	0.018	-0.102	2122	77.80	0.075	-0.291	944	83.69	0.063	-0.260
	KT625492	15885	72.84	0.076	-0.255	11193	70.39	0.087	-0.262	1473	74.75	0.015	-0.102	2122	77.71	0.075	-0.290	941	85.02	0.060	-0.291
B. cucurbitae	JN635562	15825	72.89	0.047	-0.213	11190	70.71	0.049	-0.226	1467	75.12	0.005	-0.074	2110	77.82	0.080	-0.303	946	82.35	0.014	0.042
B. diaphora	KT159730	15890	74.10	0.065	-0.241	11186	72.11	0.071	-0.243	1471	74.71	0.021	-0.113	2120	77.83	0.076	-0.306	946	85.10	0.061	-0.305
B. scutellata	KP722192	15915	72.95	0.074	-0.257	11273	70.73	0.081	-0.262	1468	74.39	0.016	-0.106	2124	77.82	0.079	-0.316	1011	84.87	0.072	-0.262
B. tau	KP711431	15687	73.28	0.060	-0.221	11273	71.40	0.059	-0.225	1471	74.92	0.015	-0.084	2119	77.73	0.073	-0.301	801	83.75	0.119	-0.132
C. capitata	AJ242872	15980	77.48	0.021	-0.185	11272	75.59	0.019	-0.178	1472	76.77	0.021	-0.094	2123	80.22	0.058	-0.267	1004	91.14	-0.010	-0.258
D. longicornis	KX345846	16253	72.33	0.101	-0.293	11208	69.40	0.105	-0.301	1481	74.81	0.052	-0.126	2129	77.17	0.087	-0.330	1343	85.26	0.146	-0.354
P. utilis	KC355248	15922	80.83	0.037	-0.175	11193	78.90	0.041	-0.170	1501	80.61	0.005	-0.100	2236	85.69	0.051	-0.300	928	90.52	0.015	-0.045

Table S3. The best partitioning scheme selected by PartitionFinder for different dataset.

Dataset	Subset Partitions	Best Model
P123RNA:<br>6 partitions (BI)	P1: (APT6_pos1, CO1_pos1, CO2_pos1, CO3_pos1, Cytb_pos1)	GTR+I+G
	P2: (APT6_pos2, CO1_pos2, CO2_pos2, CO3_pos2, Cytb_pos2, ND1_pos2, ND4L_pos2, ND4_pos2, ND5_pos2)	GTR+I+G
	P3: (APT6_pos3, ATP8_pos3, CO1_pos3, CO2_pos3, CO3_pos3, Cytb_pos3, ND2_pos3, ND3_pos3, ND6_pos3)	GTR+I+G
	P4: (ATP8_pos1, ATP8_pos2, ND1_pos1, ND2_pos1, ND2_pos2, ND3_pos1, ND3_pos2, ND4L_pos1, ND4_pos1, ND5_pos1, ND6_pos1, ND6_pos2, tRNAs)	GTR+I+G
	P5: (ND1_pos3, ND4L_pos3, ND4_pos3, ND5_pos3)	GTR+G
	P6: (12S, 16S)	GTR+I+G
P123R:<br>6 partitions (ML)	P1: (APT6_pos1, CO1_pos1, CO2_pos1, CO3_pos1, Cytb_pos1)	GTR+I+G
	P2: (APT6_pos2, CO1_pos2, CO2_pos2, CO3_pos2, Cytb_pos2, ND1_pos2, ND4L_pos2, ND4_pos2, ND5_pos2)	GTR+I+G
	P3: (APT6_pos3, ATP8_pos3, CO1_pos3, CO2_pos3,	GTR+I+G

	CO3_pos3, Cytb_pos3, ND2_pos3, ND3_pos3, ND6_pos3)	
	P4: (ATP8_pos1, ATP8_pos2, ND1_pos1, ND2_pos1, ND2_pos2, ND3_pos1, ND3_pos2, ND4L_pos1, ND4_pos1, ND5_pos1, ND6_pos1, ND6_pos2, tRNAs)	GTR+I+G
	P5: (ND1_pos3, ND4L_pos3, ND4_pos3, ND5_pos3)	GTR+I+G
	P6: (12S, 16S)	GTR+I+G
P123: 5 partitions (BI)	P1: (APT6_pos1, CO1_pos1, CO2_pos1, CO3_pos1, Cytb_pos1)	GTR+I+G
	P2: (APT6_pos2, ATP8_pos1, ATP8_pos2, CO1_pos2, CO2_pos2, CO3_pos2, Cytb_pos2, ND1_pos2, ND2_pos2, ND3_pos2, ND4L_pos2, ND4_pos2, ND5_pos2, ND6_pos2)	GTR+I+G
	P3: (APT6_pos3, ATP8_pos3, CO1_pos3, CO2_pos3, CO3_pos3, Cytb_pos3, ND2_pos3, ND3_pos3, ND6_pos3)	GTR+I+G
	P4: (ND1_pos1, ND2_pos1, ND3_pos1, ND4L_pos1, ND4_pos1, ND5_pos1, ND6_pos1)	GTR+I+G
	P5: (ND1_pos3, ND4L_pos3, ND4_pos3, ND5_pos3)	GTR+G
P123: 5 partitions (ML)	P1: (APT6_pos1, CO1_pos1, CO2_pos1, CO3_pos1, Cytb_pos1)	GTR+I+G

	P2: (APT6_pos2, CO1_pos2, CO2_pos2, CO3_pos2, Cytb_pos2, ND1_pos2, ND2_pos2, ND3_pos2, ND4L_pos2, ND4_pos2, ND5_pos2, ND6_pos2)	GTR+I+G
	P3: (APT6_pos3, ATP8_pos3, CO1_pos3, CO2_pos3, CO3_pos3, Cytb_pos3, ND2_pos3, ND3_pos3, ND6_pos3)	GTR+I+G
	P4: (ATP8_pos1, ATP8_pos2, ND1_pos1, ND2_pos1, ND3_pos1, ND4L_pos1, ND4_pos1, ND5_pos1, ND6_pos1)	GTR+I+G
	P5: (ND1_pos3, ND4L_pos3, ND4_pos3, ND5_pos3)	GTR+G
P12R: 4 partition (BI)	P1: (APT6_pos1, CO1_pos1, CO2_pos1, CO3_pos1, Cytb_pos1)	GTR+I+G
	P2: (APT6_pos2, CO1_pos2, CO2_pos2, CO3_pos2, Cytb_pos2, ND1_pos2, ND4L_pos2, ND4_pos2, ND5_pos2)	GTR+I+G
	P3: (ATP8_pos1, ATP8_pos2, ND1_pos1, ND2_pos1, ND2_pos2, ND3_pos1, ND3_pos2, ND4L_pos1, ND4_pos1, ND5_pos1, ND6_pos1, ND6_pos2, tRNAs)	GTR+I+G
	P4: (12S, 16S)	GTR+I+G
P12R: 4 partition (ML)	P1: (APT6_pos1, CO1_pos1, CO2_pos1, CO3_pos1, Cytb_pos1)	GTR+I+G

	P2: (APT6_pos2, CO1_pos2, CO2_pos2, CO3_pos2, Cytb_pos2, ND1_pos2, ND4L_pos2, ND4_pos2, ND5_pos2)	GTR+I+G
	P3: (ATP8_pos1, ATP8_pos2, ND1_pos1, ND2_pos1, ND2_pos2, ND3_pos1, ND3_pos2, ND4L_pos1, ND4_pos1, ND5_pos1, ND6_pos1, ND6_pos2, tRNAs)	GTR+I+G
	P4: (12S, 16S)	GTR+I+G
P12: 3 partition (BI)	P1: (APT6_pos1, CO1_pos1, CO2_pos1, CO3_pos1, Cytb_pos1)	GTR+I+G
	P2: (APT6_pos2, ATP8_pos1, ATP8_pos2, CO1_pos2, CO2_pos2, CO3_pos2, Cytb_pos2, ND1_pos2, ND2_pos2, ND3_pos2, ND4L_pos2, ND4_pos2, ND5_pos2, ND6_pos2)	GTR+I+G
	P3: (ND1_pos1, ND2_pos1, ND3_pos1, ND4L_pos1, ND4_pos1, ND5_pos1, ND6_pos1)	GTR+I+G
P12: 3 partition (ML)	P1: (APT6_pos1, CO1_pos1, CO2_pos1, CO3_pos1, Cytb_pos1)	GTR+I+G
	P2: (APT6_pos2, ATP8_pos1, ATP8_pos2, CO1_pos2, CO2_pos2, CO3_pos2, Cytb_pos2, ND1_pos2, ND2_pos2, ND3_pos2, ND4L_pos2, ND4_pos2, ND5_pos2, ND6_pos2)	GTR+I+G

	P3: (ND1_pos1, ND2_pos1, ND3_pos1, ND4L_pos1, ND4_pos1, ND5_pos1, ND6_pos1)	GTR+I+G
